# Association of Statins with Sensory and Autonomic Ganglionopathy

**DOI:** 10.3389/fnagi.2015.00191

**Published:** 2015-10-07

**Authors:** Peter Novak, Daniela A. Pimentel, Banu Sundar, Majaz Moonis, Lan Qin, Vera Novak

**Affiliations:** ^1^Department of Neurology, University of Massachusetts Medical School, Worcester, MA, USA; ^2^Department of Neurology, Beth Israel Deaconess Medical Center, Harvard Medical School, Boston, MA, USA

**Keywords:** statins, small-fiber neuropathy, ganglionopathy, neuronopathy, statin toxicity

## Abstract

**Objective:**

To examine if statins have an effect on small nerve fibers.

**Methods:**

This retrospective study evaluated the effect of statins in pure small-fiber neuropathy (SFN). Outcome measures were symptom scales (numbness, tingling, and autonomic symptoms), skin biopsies assessing epidermal nerve fiber density (ENFD), sweat gland nerve fiber density (SGNFD), and quantitative autonomic testing.

**Results:**

One hundred and sixty participants with pure SFN were identified. Eighty participants (women/men, age ± SD 33/47, 68.1 ± 11.6 years old) were on statins for 53.5 ± 28.7 months to treat dyslipidemia and they were age and gender matched with 80 participants (33/47, 68.1 ± 9.5) that were off statins. ANOVA showed reduced ENFD/SGNFD at the proximal leg in the statin group [(count/mm) 8.3 ± 3.6/51.3 ± 14.2] compared to the off statin group (10.4 ± 3.8, *p* = 0.0008/56.4 ± 12.7, *p* = 0.018). There was no difference in ENFD/SGNFD at the distal leg in the statin group (4.9 ± 3.2/39.8 ± 15.7) compared to the off statin group (5.9 ± 3.4, *p* = 0.067/41.8 ± 15.9, *p* = 0.426). Statins did not affect symptom scales and the outcome of autonomic testing.

**Conclusion:**

Statin use is associated with degeneration of sensory and autonomic fibers. The pattern of abnormalities, e.g., degeneration of proximal while sparing of distal fibers, is consistent with a non-length-dependent process with lesions in the dorsal root and the autonomic ganglia. The statin-associated sensory and autonomic ganglionopathy is mild.

## Introduction

Statins, the 3-hydroxy-3-methylglutaryl coenzyme A (HMG-CoA) reductase inhibitors, are among the most prescribed medications worldwide (Lewington et al., 2007). Statins lower cholesterol levels, which can decrease the risk of cardiovascular and cerebrovascular disorders. Statins also possess cholesterol-independent pleotrophic effects that include improved endothelium-dependent vasodilation, inflammation, lipid peroxidation, and oxidative stress reduction (Willey and Elkind, 2010; Millar and Floras, 2014).

While the association of statins with liver (5–10%) and muscle toxicity (<1% of statin users) is well defined, their association with the function of the peripheral nervous system remains incompletely understood (Gaist et al., 2002; Anderson et al., 2005; Kiortsis et al., 2007; Argov, 2015). On rare occasions (1 case for every 2200 statin patients-years), statins can induce large fiber neuropathy that may be related to inhibition of ubiquinone, a key mitochondrial respiratory chain enzyme (Kiortsis et al., 2007), or to excessive depletion of cholesterol of nerve membranes (Gaist et al., 2002). Knowledge about the association of statins with small-fiber neuropathy (SFN) is limited. One report described three patients in which statins were associated with sensory symptoms and abnormal sympathetic skin responses (Lo et al., 2003), and another case-report described a patient with abnormal quantitative sudomotor reflex test (Boger et al., 2011). Both reports attributed abnormal findings to statin-induced SFN. On the other hand, statins may have beneficial effects on the peripheral nervous system including small fibers since they may improve diabetic polyneuropathy (Hernández-Ojeda et al., 2014) or autonomic functions (Millar and Floras, 2014), presumably via pleotropic effects.

The goal of this study was to analyze the long-term effect of statins in patients with pure SFN.

## Materials and Methods

### Standard protocol approvals, registrations, and patient consents

The study was approved by the Institutional Review Board of the University of Massachusetts Medical School.

### Patient selection

This was a retrospective single center study that included patients who were referred for SFN evaluation at the Autonomic Laboratory, University of Massachusetts Medical School, between 2009 and 2015. All subjects were reviewed for symptoms and signs suggestive of SFN from electronic case records.

### Small-fiber neuropathy

Small-fiber neuropathy affects sensory and autonomic small fibers resulting in a combination of sensory and autonomic symptoms (Novak et al., 2001; Devigili et al., 2008; Hovaguimian and Gibbons, 2011). Typical sensory symptoms include distal limb burning, prickling, stabbing discomfort, numbness or tingling. SFN-associated autonomic symptoms include excessive coldness, discoloration, erythromelalgia, hyper- or hypohydrosis, orthostatic intolerance, and enteric or urinary symptoms (Novak et al., 2001; Devigili et al., 2008). Multiple disorders are associated with SFN including impaired glucose tolerance, metabolic syndrome, thyroid dysfunction, sarcoidosis, vitamin B12 deficiency, HIV, neurotoxic medications including chemotherapeutic and antiretroviral agents, celiac disease, paraneoplastic syndromes, alcohol abuse, Sjogren syndrome, elevated triglycerides, and paraproteinemias (McArthur et al., 1998; Novak et al., 2001; Low et al., 2006; Devigili et al., 2008; Hovaguimian and Gibbons, 2011). In pure SFN which represent about 50% of all SFN cases, no cause can be identified.

### Symptom scales

Sensory and autonomic assessments were done at an autonomic laboratory. Sensory evaluations included self-reported pain intensity using a 0–10 numerical rating scale that is part of the NIH Toolbox (Cook et al., 2013) and numbness or tingling scale using a 0–4 numerical grading (none–very rare/mild–frequent–continuous). The Scale for Outcomes in Parkinson’s Disease-Autonomic [SCOPA-AUT (Visser et al., 2004)] has been used to evaluate autonomic symptoms. SCOPA-AUT consists of 25 items assessing dysfunction in the gastrointestinal, urinary, cardiovascular, thermoregulatory, pupillomotor and sexual domains. Each item is scored from 0 (no symptoms), 1 (rare), 2 (frequent) to 3 (severe).

### Skin biopsies

Skin biopsies were performed for the assessment of epidermal and sweat gland nerve fibers. Skin biopsy can reliably demonstrate loss of small epidermal fibers with a sensitivity and specificity equal to 88 and 88.8%, respectively for the detection of SFN (Devigili et al., 2008). Skin biopsies were performed following the recommended standards (McArthur et al., 1998; Devigili et al., 2008; Lauria et al., 2010; Hovaguimian and Gibbons, 2011). Skin biopsies were obtained from the proximal thigh 20 cm distal to the iliac spine and at the calf (10 cm above the lateral malleolus) using a 3-mm circular disposable punch tool. Samples were immediately transferred into 2% paraformaldehyde-lysine-periodic acid fixative. Sample processing was done at commercial laboratory in Therapath, New York, NY, USA. Therapath is a CLIA-certified laboratory and is accredited by College of American Pathologists. Samples were immunoperoxidase-stained with the panaxonal marker PGP 9.5. Linear epidermal nerve fiber density (ENFD) was obtained using bright light microscopy following the guidelines of the European Federation of the Neurological Societies (Lauria et al., 2010). ENFD was obtained in three or more sections and calculated as the number of nerve fibers per length (density/mm) crossing the dermal–epidermal junctions. Secondary branching was excluded from the analysis. Sweat gland nerve fiber density (SGNFD) was obtained from the same tissue sections also stained for PGP 9.5. Sweat glands were captured using an Olympus photomicrograph and analyzed according to the method described previously (Gibbons et al., 2009). All fiber counts were done by a board certified neuropathologist that was blinded to the use of statins.

### Quantitative autonomic testing

All subjects referred for SFN underwent standardized autonomic testing (Low et al., 2006; Novak, 2011) described in detail previously (Novak, 2011, 2015). Cardiovascular autonomic reflex tests included deep breathing, the Valsalva maneuver and tilt-table test. Postganglionic sudomotor functions were assessed by quantitative sudomotor axon reflex test (QSART) in the forearm, proximal leg, distal leg, and foot, using the Q-Sweat machine (WR Medical Electronics, Stillwater, MN, USA).

### Grading of tests

The standardized functional cardiovascular reflex autonomic tests (deep breathing, Valsalva maneuver, and tilt test) and skin biopsies (ENFD and SGNFD) were graded using Quantitative Scale for Grading of Cardiovascular Autonomic Reflex Tests and Small Fibers from Skin Biopsies (QASAT) (Novak, 2015). QASAT grades cardiovascular tests, QSART, ENFD, and SGNFD. We used QASAT-heart rate variability as a marker of cardiovagal activity and QASAT-adrenergic failure (obtained as combination of the Valsalva maneuver and orthostatic hypotension score) as a marker of sympathetic adrenergic failure. QASAT assigns a number to each test result that is proportional to the severity of findings. Normal test results correspond to 0, while abnormal results are >0. Higher scores indicate more severe impairment. QASAT uses age and gender adjusted normative data (if applicable) and scores are generated automatically.

### Definition of pure SFN

The heterogeneity of SFN causes can mask the effect of statins, therefore pure SFN was selected for this study. Available SFN criteria use either sensory (Devigili et al., 2008) or autonomic (Low et al., 2006) evaluations.

Devigili et al. (2008) defined SFN by combining signs suggestive of SFN (pinprick and thermal sensory loss, allodynia, and hyperalgesia) and reduced ENFD, the latter being the most useful single test with a diagnostic efficiency of 88.4%. Low et al. (2006) used abnormal sudomotor evaluations in SFN detection with a sensitivity equal to 98%. Pure SFN affects both sensory and autonomic fibers and therefore we defined pure SFN by the combination of sensory and autonomic evaluations.

In this study, pure SFN was defined by the following criteria: (1) presence of sensory and/or autonomic symptoms suggestive of SFN; (2) QASAT score >0. An abnormality in any single QASAT section will increase the score above 0 including a single abnormality in EFND, SGFND, or any abnormality in cardiovascular autonomic functional testing; (3) all secondary causes of SFN have to be ruled out. Medical records were screened for the following secondary causes of SFN: abnormal glucose metabolism, large fiber neuropathy, Parkinson disease, atypical parkinsonism, multiple system atrophy, history of heavy alcohol exposure, B12 deficiency, folate deficiency, thyroid disease, hepatitis C, HIV infection, exposure to chemotherapy, use of neurotoxic medication, cancer, Sjogren syndrome, any other comorbid condition or use of medication reported to be associated with SFN. Abnormal glucose metabolism was defined as hemoglobin A1C, fasting glucose or glucose tolerance test results indicative of diabetes, prediabetes, or abnormal glucose tolerance.

### Study inclusion criteria

The inclusion criteria were (1) patients older than 17 years; (2) availability of clinical records; (3) completed autonomic testing; and (3) results of autonomic testing consistent with pure SFN. To be included in the study, all the inclusion criteria had to be met.

We also collected statin type, dose, duration of treatment, and cumulative dose. The one dose equivalent was defined as atorvastatin 10 mg/day, rosuvastatin 5 mg/day, lovastatin 40 mg/day, pravastatin 40 mg/day, or simvastatin 20 mg/day (Anderson et al., 2005; Wu et al., 2015).

### Statistical analysis

One-way ANOVA was used to compare the groups with and without statins. The relationships between continuous variables were obtained using Pearson correlation coefficient (*r*). All statistical analyses were performed using JMP 12 (Cary, NC, USA) statistical software.

## Results

**Figure 1:**
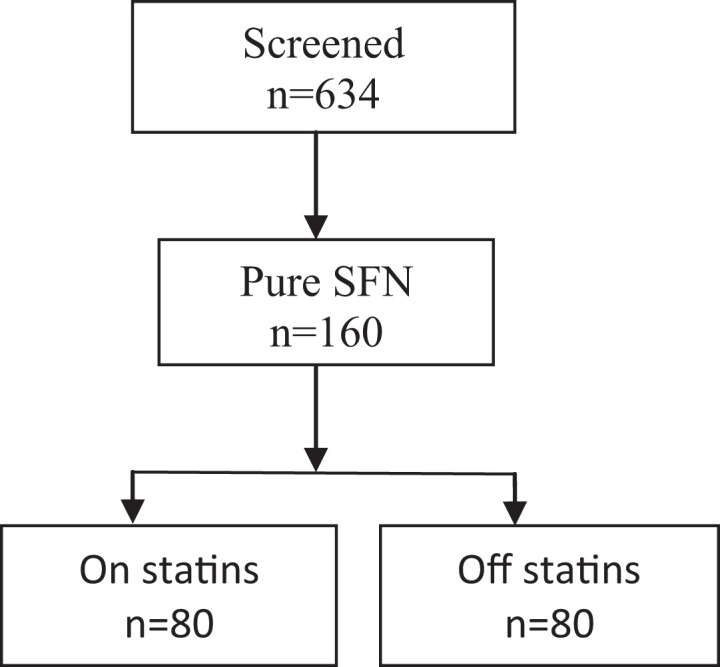
**Participants’ flow diagram**.

From our database we screened 634 participants referred for evaluation of SFN. One hundred and sixty participants fulfilled inclusion/exclusion criteria for pure SFN, 80 were treated with statins (Figure 1). Both on and off statin groups were age and gender matched. Participant’s characteristics and the effects of statins are summarized in Table 1.

**Table 1:** **Characteristics of study participants**.

Variable		Participants		*P* value
		Off statins	On statins	
Number of participants (f/m)		80	80	1
Age (years)		68.1 ± 9.5	68.1 ± 11.6	0.99
Gender (f/m)		33/47	33/47	1
BMI (kg/m^2^)		26.9 ± 4.6	26.9 ± 6.1	0.95
Statins	Dose equivalent	0	2.5 ± 2.2	N/A
	Treatment duration (months)	0	53.5 ± 28.7	N/A
	Cumulative dose	0	142.1 ± 169.5	N/A
ENFD-calf (count/mm)		5.9 ± 3.4	4.9 ± 3.2	0.067
ENFD-thigh (count/mm)		10.4 ± 3.8	8.3 ± 3.6	0.0008
SGNFD-calf (count/mm)		41.8 ± 15.9	39.8 ± 15.7	0.426
SGNFD-thigh (count/mm)		56.4 ± 12.7	51.3 ± 14.2	0.018
SCOPA-AUT		15.8 ± 11.6	15.9 ± 14.4	0.93
Pain scale		3.1 ± 1.64	3.1 ± 1.75	0.96
Numbness scale		1.79 ± 0.7	1.75 ± 0.76	0.619
Pain duration (months)		4.4 ± 4.8	4.8 ± 6.1	0.67
Numbness duration (months)		4.9 ± 5.2	4.1 ± 4.6	0.65
QASAT-cardiovagal		1.5 ± 1.0	1.4 ± 1.2	0.578
QASAT-adrenergic failure		4.3 ± 4.3	3.9 ± 3.9	0.52

*Means presented as ±SD*.

*ENFD, epidermal nerve fiber density; SGNFD, sweat gland nerve fiber density*.

**Figure 2:**
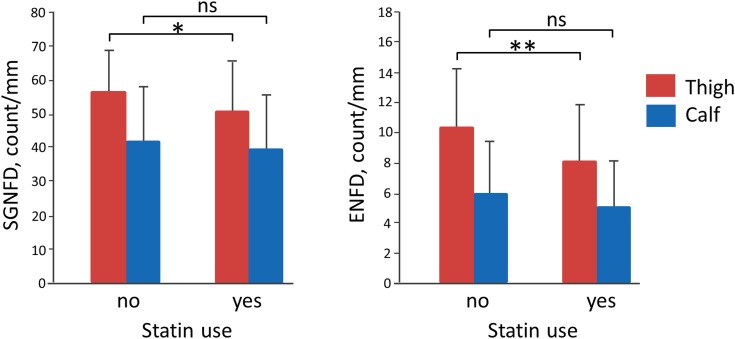
**Effect of statins**. ENFD, epidermal nerve fiber density; SGNFD, sweat gland nerve fiber density; ns, non-significant. *Significant, *p* = 0.018 and **significant, *p* = 0.0008.

When comparing statin users with no-users using ANOVA, the statin group had reduced ENFD and SGNFD at the thigh but not at the calf (Table 1; Figure 2). All other comparisons were not significant. There was no difference in autonomic testing (QASAT-HRV, QASAT-adrenergic failure) scores between both groups. There was no significant difference in subjective scales (pain, numbness, and SCOPA-AUT) between both groups.

Correlation analysis using the Pearson coefficient showed a significant correlation between the cumulative dose of statins and thigh ENFD (*r* = −0.21, *p* = 0.0065). All other correlations between the cumulative dose of statins and continuous variables were not significant. These variables were SGFND at the thigh (*r* = −0.11, *p* = 0.16), SGFND at the calf (*r* = −0.01, *p* = 0.88), ENFD at the calf (*r* = −0.03, *p* = 0.65), SCOPA-AUT (*r* = −0.01, 0.83), the pain scale (*r* = 0.06, *p* = 0.54), and the numbness scale (*r* = 0.03, *p* = 0.68).

## Discussion

Our main result is the finding of an association between statins and small nerve fiber degeneration. Statin users had both sensory and autonomic sudomotor fibers reduced at the thigh. The cumulative statin dose correlated inversely with the sensory fiber density at the thigh. Furthermore, symptom scales were not affected by statins. Participants treated with statins did not report more sensory and autonomic symptoms compared to the statin non-users.

The proximal, e.g., at the thigh, degeneration of sensory and autonomic axons with sparing of distal axons indicates a non-length-dependent process. This pattern can be explained by simultaneous lesions in the dorsal root ganglia and the autonomic ganglia causing sensory and autonomic abnormalities, respectively (Sghirlanzoni et al., 2005; Gorson et al., 2008; Gemignani et al., 2010). Then our findings can be interpreted that statin use is associated with sensory and autonomic ganglionopathy.

Sensory and autonomic ganglionopathy, also called sensory and autonomic neuronopathy, although rare, can be associated with variety of conditions including infection, inflammation, autoimmunity, critical illness, glucose metabolism abnormalities, Sjogren syndrome, paraneoplastic, or toxic effects (Satake et al., 1998; Sumner et al., 2003; Sghirlanzoni et al., 2005; Gorson et al., 2008; Chai and Logigian, 2010; Gemignani et al., 2010; Koike and Sobue, 2013; Latronico et al., 2013; Birnbaum and Bingham, 2014). These ganglionopathies are typically acute and associated with severe neuropathic dysfunction, although subacute and chronic variants also have been reported. In our cohort, these conditions were ruled out.

The statin-induced ganglionopathy is most likely mild for several reasons. First, sensory and autonomic symptoms were not different in statin users compared to the non-users even it can even be argued that sensory symptoms, in general, do not correlate with duration and severity of SFN (Devigili et al., 2008). Second, the mean proximal ENFD loss was around 20% and the mean proximal SGFND loss was around 9% in statin users. Using the QASAT grading instrument (Novak, 2015), that amount of small fiber loss is approximately equal to one abnormality range using the normal, mildly-moderately-severely-markedly abnormal grading system. Since the baseline fiber density before the onset of statin treatment is unknown, we can only assume mild worsening of the underlying fiber density.

There are several possible mechanisms explaining statin-induced ganglionopathy. The impairment of energy and protein metabolism, and the induction of necrosis or apoptosis was associated with statin-induced myopathy (Gaist et al., 2002; Kiortsis et al., 2007; Lewington et al., 2007; Willey and Elkind, 2010; Millar and Floras, 2014; Argov, 2015). The statin-induced degeneration of the peripheral ganglia may involve myelin sheath damage of small myelinated fibers caused by the inhibition of HMG-CoA reductase, impairing the conversion of HMG-CoA to mevalonate, which is crucial for the production of cholesterol (Jones et al., 2014; Norata et al., 2014). Mevalonate is also a precursor of farnesyl pyrophosphate (FPP), essential for the production of ubiquinone and thus, for the production of ATP by the electron transport chain (Jones et al., 2014; Norata et al., 2014; Wilkinson et al., 2014; Patel et al., 2015). FPP depletion can also impair the production of geranylgeranyl pyrophosphate (Jones et al., 2014; Norata et al., 2014). Both molecules contribute to the posttranslational modification of specific intracellular proteins (such as Rho, Ras, Rab, and nuclear lamins) that if dysfunctional, can lead to apoptosis (Jones et al., 2014; Norata et al., 2014). In summary, several mechanisms may underlie the toxic effect of statins as well as genetic predisposition.

There are several shortcomings of our study. The study has retrospective character, various doses of statins among participants, use of particular type statins, and other biases. Therefore, it is possible that our study was not sensitive enough to detect a symptomatic effect of statins. Furthermore, we did not evaluate markers of abnormal glucose metabolism, the most common cause of the SFN (Hovaguimian and Gibbons, 2011), although we did exclude patients with history of abnormal glucose metabolism. Nevertheless, abnormal glucose metabolism is usually associated with the length-dependent (distal) SFN (Polydefkis et al., 2003; Hovaguimian and Gibbons, 2011) and not with the non-length-dependent pattern that was observed in our study. Hence, it is unlikely that abnormal glucose metabolism contributed to our results.

In conclusion, our study indicates that statins have neuropathic effects. The distribution of abnormalities and the severity of symptoms are most consistent with mild, likely subclinical, sensory, and autonomic ganglionopathy.

## Author Contributions

PN and LQ collected data. PN and VN were responsible for statistical analysis. PN wrote the first draft of the paper. All authors reviewed the study findings, the paper and approved the paper. PN performed statistical analysis.

## Conflict of Interest Statement

The authors declare that the research was conducted in the absence of any commercial or financial relationships that could be construed as a potential conflict of interest.

## Funding

The study was funded by Department of Neurology, University of Massachusetts Medical School, Worcester, MA, USA. The authors thank Shane Stanek and Steve Smajkiewicz for their help in data collection.

## Abbreviations

ENFD, epidermal nerve fiber density; FPP, farnesyl pyrophosphate; HMG-CoA, the 3-hydroxy-3-methylglutaryl coenzyme A; QASAT, quantitative scale for grading of cardiovascular autonomic reflex tests and small fibers from skin biopsies; SCOPA-AUT, the scales for outcomes in Parkinson’s disease-autonomic; SFN, small-fiber neuropathy; SGNFD, sweat gland nerve fiber density.
